# Update on Pharmacological Treatment for Comorbid Major Depressive and Alcohol Use Disorders: The Role of Extended-release Trazodone

**DOI:** 10.2174/1570159X21666230403080624

**Published:** 2023-09-01

**Authors:** Marco Di Nicola, Maria Pepe, Isabella Panaccione, Lorenzo Moccia, Luigi Janiri, Gabriele Sani

**Affiliations:** 1 Department of Psychiatry, Fondazione Policlinico Universitario Agostino Gemelli IRCCS, Rome, Italy;; 2 Department of Neuroscience, Section of Psychiatry, Università Cattolica del Sacro Cuore, Rome, Italy;; 3 Mental Health Department, ASL Roma 1, Rome, Italy

**Keywords:** Trazodone, comorbidity, depression, alcoholism, antidepressants, personalized medicine

## Abstract

**Background:**

Major Depressive Disorder (MDD) and Alcohol Use Disorder (AUD) are major public health concerns because of their high prevalence and clinical and functional severity. MDD and AUD commonly co-occur, but effective therapeutic approaches for comorbidity are still scarce. Available evidence on selective serotonin reuptake inhibitors and tricyclic antidepressants held mixed results, and further pharmacological categories have been less investigated. Trazodone is an approved antidepressant drug for adults and has shown efficacy on symptoms like anxiety and insomnia observed in AUD patients as well. Thus, this study aims to evaluate the effect of extended-release trazodone on clinical and functional features in MDD + AUD subjects.

**Methods:**

One hundred MDD + AUD outpatients were retrospectively evaluated at 1, 3, and 6 months of treatment with extended-release trazodone (150-300 mg/day, flexibly dosed). Improvement in depressive symptoms was the primary outcome measure. Changes in anxiety, sleep, functioning, quality of life, clinical global severity, and alcohol craving were also investigated.

**Results:**

Trazodone reduced depressive symptoms (*p <* 0.001) with 54.5% remission at the endpoint. Similar improvements were observed in all secondary outcomes, including anxiety, sleep alterations, and craving (*p <* 0.001). Only mild side effects were reported and disappeared over time.

**Conclusion:**

Extended-release trazodone displayed good antidepressant properties in MDD + AUD patients, ameliorating overall symptomatology, functioning, and quality of life, with a good safety/ tolerability profile. Further, it significantly improved sleep disturbances and craving symptoms, which are associated with drinking relapse and worse outcomes. Therefore, trazodone might represent a promising pharmacological option for MDD + AUD patients.

## 1 INTRODUCTION

Major Depressive Disorder (MDD) and Alcohol Use Disorder (AUD) are highly prevalent diseases and are associated with increased rates of morbidity, disability, and mortality. MDD affects about 300 million people worldwide and is expected to become the leading cause of disability by 2030 [1]. Similarly, more than 100 million people can be diagnosed with AUD, although only a minority seeks help [2].

MDD and AUD are frequently comorbid, with MDD being the psychiatric disorder most found in subjects with AUD, and the presence of either condition doubles the risk of developing the other [3, 4]. The Diagnostic and Statistical Manual of Mental Disorders, Fifth Edition (DSM-5) differentiates primary depression from substance-induced disorders; thus, a diagnosis of depression during active drinking or alcohol withdrawal should be made with caution. Accordingly, current guidelines indicate that in MDD + AUD patients, antidepressant treatment should be started only after about 4-weeks of abstinence from alcohol, a period that allows diagnosing an independent depressive disorder with more confidence. Nonetheless, it has been reported that more than ¼ of AUD patients experienced a substance-induced depressive episode in their lifetime [5]. It is also evident that substance-induced depression itself increases the risk for independent depressive disorders. Also, a significant rate of cases initially diagnosed as “substance-induced” are later reclassified as “primary” depression, thus encouraging to consider antidepressant treatment in these patients [6].

Repeated evidence highlights the association of comorbid MDD + AUD with increased symptom severity, worse response to treatments, and detrimental clinical and functional outcomes [6, 7]. A recent study has shown that subjects with depression/anxiety have a particularly high risk of alcohol misuse immediately before starting the antidepressant therapy [8], which might reflect the worsening of substance use problems alongside the worsening of mood/anxiety symptoms. In addition, alcohol use negatively affects the frequency and duration of depressive episodes and is associated with a higher suicide risk [9]. Moreover, among patients with AUD, depressive symptoms can increase the risk of both mood-induced episodes of heavy drinking and relapse during early abstinence, while individuals who never had depressive or anxiety disorders seem to be more likely to achieve remission [10]. Additionally, specific features, *i.e*., anxiety and insomnia, seem to be particularly associated with a higher risk of both mood and drinking relapses, and could benefit from targeted interventions [11, 12]. However, despite the significance of MDD + AUD comorbidity, these patients tend to be excluded from registration trials, and existing guidelines mostly focus on either disorder.

### 1.1 Management of MDD + AUD: Evidence from the Literature

Several meta-analyses and systematic reviews summarized the available evidence on the efficacy of both psychopharmacological and psychosocial interventions more commonly employed in clinical practice for addressing MDD + AUD.

A meta-analysis of placebo-controlled randomized clinical trials (RCTs) showed that certain antidepressants (*i.e*., tricyclic antidepressants (TCAs), nefazodone) were more effective than placebo in treating depression in comorbid MDD + AUD patients [13]. A subsequent systematic review assessed the risks and benefits of antidepressants in MDD + AUD patients by investigating clinical trials comparing antidepressants, alone or in association with other drugs or psychosocial interventions (or both) *vs.* placebo, no treatment, and other pharmacological or psychosocial interventions [14]. Results from this study reported some efficacy of several classes of antidepressants in improving both depressive symptoms and certain AUD-related features, such as the number of drinks per drinking day and the percentage of patients maintaining abstinence [14]. Although results were not always consistent across the studies, subsequent reviews supported the use of psychopharmacotherapy for comorbid MDD + AUD and highlighted the importance of concurrently treating both conditions to achieve better clinical outcomes [6, 15, 16]. A bayesian network meta-analysis of RCTs on pharmacological treatments of MDD + AUD [9] aimed at comparing and ranking the efficacy of current treatments on both mood- and AUD-related outcomes. Results have shown that monotherapy with either antidepressants or anti-craving drugs was insufficient in addressing concurrent symptoms, supporting the need for a combined therapy despite the increased tolerability concerns. A network meta-analysis further confirmed the abovementioned findings, suggesting that treatment with TCAs and Selective Serotonin Reuptake Inhibitors (SSRIs) improves depressive symptoms and functional status in patients dually diagnosed, while effects on drinking outcomes appeared to be less relevant [17]. By contrast, a recent bayesian meta-analysis on 64 RCTs with 6128 participants found that SSRIs (specifically fluoxetine) not only improved depressive symptoms in individuals with AUD but also facilitated abstinence and reduced alcohol use and craving independently of dosage or treatment length [18].

Recent clinical studies have shown as well that SSRIs improved both depressive symptoms and craving measures [19] and confirmed the efficacy of fluoxetine, fluvoxamine, and citalopram in treating depression in MDD + AUD patients, with statistically significant differences related to the presence of CYP polymorphisms [20-22]. However, these findings were limited by the small sample size, the heterogeneity of inclusion criteria, and the lack of a control group.

So far, investigations on MDD + AUD have focused mainly on the effects of SSRIs and different classes of antidepressants have been less investigated.

Two recent, preliminary, real-world studies highlighted the effectiveness of vortioxetine in improving depressive symptoms in MDD + AUD patients, with also beneficial effects in reducing alcohol consumption [23, 24]. New antidepressants with glutamatergic activity (*i.e*., ketamine, esketamine), recently approved for treatment-resistant depression, are currently being tested for further conditions [25]. A case series on comorbid MDD + AUD patients described that treatment with naltrexone and ketamine was associated with improvement in depressive symptoms, as well as in alcohol craving and consumption [26]. However, these observations will need to be replicated in much larger studies with different designs to provide informative results.

### 1.2 Limitations and ‘Unmet Needs’ of Systematic Reviews, Meta-analyses, and RCTs

Despite the substantial efforts to systematically analyze the available evidence and the most recent clinical trials, to date, results are not consistent enough to inform guideline recommendations. Several reasons could be argued.

First, the relatively small number of clinical studies on comorbid MDD + AUD patients, especially RCTs. Second, the diagnosis of comorbid MDD and AUD might be challenging because of overlapping symptoms and shared features, like the depressant effects of alcohol, insomnia, and psychomotor agitation. Diagnostic criteria for AUD varied between DSM-IV and DSM-5, and depressive disorders include several conditions with different features. Also, clinical studies included in systematic analyses differ in terms of enrolling MDD + AUD patients with active drinking or recently sober. All these factors might have contributed to increased heterogeneity of the sample within studies and limited the consistency of results. Third, most clinical trials compared the effects of antidepressants *vs*. placebo, and only a few studies with small sample sizes included active comparators so it is currently not possible to define whether specific compounds might prove superior efficacy. Finally, there is a substantial disproportion in the number of available studies that employed TCAs and SSRIs, while molecules with different mechanisms of action have been less investigated.

Despite these limitations, systematic studies agree that antidepressants may be useful therapeutic options in people with co-occurring depressive and alcohol-use disorders.

### 1.3 New Potential Perspectives for Treatment

Given the high prevalence of comorbid MDD + AUD, its detrimental outcomes, the burden on the healthcare system, and the relevance of unmet needs, defining therapeutic approaches that effectively address both conditions is urgent.

Neuromodulation interventions have been increasingly studied as treatment options for several psychiatric disorders. Repetitive transcranial magnetic stimulation (rTMS) and transcranial direct current stimulation (tDCS) are non‐invasive and safe techniques used to inhibit or promote local neural activity in specific cortical areas that have been mainly investigated in mood and obsessive-compulsive disorders [27, 28]. Repetitive TMS is an evidence-based treatment for MDD [29] and promising evidence has been raising in the addiction field, especially for the treatment of substance use disorders [30]. Findings from a real-world study on subjects with substance and alcohol use disorders show a significant and rapid reduction of craving following 5 consecutive days of tDCS sessions, alongside improvement in depressive and anxious symptoms, consistent with previous studies [30, 31]. Such results, together with the wider experience achieved on depressive disorders, suggest that synergistic approaches combining pharmacological treatments, psychotherapies, and neuromodulation strategies might represent valid interventions to address MDD + AUD comorbidity.

Further insights on currently available pharmacological options also appear crucial. An interesting meta-analysis highlighted the efficacy of the Serotonin Antagonist and Reuptake Inhibitor (SARI) nefazodone in treating depression in patients with comorbid AUD [13]. Also, subsequent results [9] show that SARIs were second only to disulfiram in increasing abstinent days in MDD + AUD patients while showing similar efficacy to SSRIs in reducing depressive symptoms. However, these promising observations derived from a small number of studies, and additional data could help in clarifying the therapeutic potential of these compounds.

Trazodone is a SARI antidepressant approved in most countries for the treatment of depressive disorders in adults [32, 33], it can be administered in several routes (*i.e*., oral, intravenous, intramuscular) and displays multiple mechanisms of action, including relevant serotonin 5-HT2A and α1-adrenergic receptor antagonism, serotonin reuptake inhibition, and weak antihistamine or histamine H1 receptor inverse agonism [32]. Immediate-release (IR) and extended-release (ER) tablets are available as oral formulations with distinct pharmacodynamic and pharmacokinetic aspects that possibly explain the different effects on specific symptoms [34, 35].

Because of its therapeutic flexibility, the off-label use of trazodone is common in clinical practice for many other conditions, including anxiety and sleep problems [36]. A recent systematic review with meta-analysis on insomnia disorders reported the beneficial effects of trazodone and its ability to optimize the internal structure of sleep, possibly associated with the blockade of 5-HT2 serotonin, H1 histamine, and α1 adaptive receptors. Favorable effects on several polysomnographic parameters have been reported: increased total sleep time, reduced latency to onset of persistent sleep and number of awakenings [37], restored sleep continuity, slow-wave sleep, and REM phases, which were demonstrated to be specifically altered in patients with depression [38]. Interestingly, such sedative-hypnotic mechanisms and effects on sleep architecture have been hypothesized to give trazodone also a role in post-traumatic stress disorder (PTSD). A recent report on low dosages of ER formulations combined with a SSRI antidepressant found that PTSD symptoms, especially nightmares, improved until remission and, likely, not only because of the potentiation of SSRIs action in prefrontal cortex areas implied in PTSD pathophysiology [39].

Anxiety and sleep problems are usually relevant in AUD and are associated with an increased risk of drinking relapses [40]. Preliminary reports have shown that low doses of trazodone decreased craving for alcohol, as well as depressive and anxiety symptoms, in a small sample of AUD patients [41]. Taken together, these observations suggest that trazodone might represent a useful pharmacological option in the treatment of MDD + AUD patients, but data on its effectiveness in this clinical population are still scarce. Therefore, the study aims to retrospectively investigate the effect of extended-release trazodone on depressive symptoms in adults with comorbid MDD and AUD. In addition to assessing mood, we were interested in evaluating anxiety and sleep, measures of craving, overall functioning, and quality of life.

## 2 MATERIALS AND METHODS

### 2.1 Participants

We retrospectively evaluated MDD + AUD outpatients, consecutively referring to the Department of Psychiatry of the Fondazione Policlinico Universitario “A. Gemelli” IRCCS in Rome, between January 2017 and December 2019. Participants were screened for MDD and AUD according to DSM-5 criteria [42] and diagnoses confirmed by the Italian version of the Structured Clinical Interview for DSM-5 Disorders Clinician Version (SCID-5-CV) [43].

Subjects were considered eligible for the study if having a Montgomery-Åsberg Depression Rating Scale (MADRS) total score ≥ 26, if the current depressive episode lasted for ≥3 months, and if abstinent from at least 4 weeks before starting pharmacological treatment. Further inclusion criteria were age 18 to 65 years, at least 8 years of education, and fluency in spoken and written Italian. Exclusion criteria were psychotic features, current substance use disorder (SUD) except for nicotine dependence, major medical disorders or organic brain syndromes, neurocognitive disorders, or significant cognitive impairment based on a Mini-Mental State Examination (MMSE) score < 26 [44].

Patients were prescribed extended-release trazodone, both prolonged-release (PR) 75-150 mg tablets and once-a-day (OAD) 150-300 mg formulations, at a flexible dose of 150-300 mg/day (based on clinical evaluation) as part of an integrated therapeutic-rehabilitation program. Prescription of either PR or OAD tablets had been made by clinicians as per routine clinical practice according to patients’ clinical picture, individual needs, and tolerability. All participants were provided continuous psychosocial support throughout the treatment period. Subjects at any time undergoing relapse to heavy drinking, *i.e*., either consuming five (four for women) or more standard drinks on a single occasion or drinking on five or more days in one week after a period of sustained abstinence [45] or discontinuing the therapeutic-rehabilitation program, were considered dropouts and excluded from subsequent evaluations. Data were obtained from measurements and assessments performed at baseline and after 1, 3, and 6 months of treatment (endpoint).

The study protocol was conducted following the Good Clinical Practice guidelines and the Declaration of Helsinki (1964) and subsequent revisions and was approved by the Ethics Committee of the Fondazione Policlinico Universitario “A. Gemelli” IRCCS, Università Cattolica del Sacro Cuore, Rome (Italy).

### 2.2 Procedures and Assessment

Mood symptoms were assessed using MADRS at baseline, 1 month, 3 months, and 6 months [46]. Patients were considered responders when obtaining an improvement of at least 50% of MADRS baseline scores and remitters when achieving a total score ≤ 7 at the endpoint, while mood relapses were defined as a new worsening of depressive symptoms (MADRS total score ≥ 18) after initial improvement.

Anxiety levels were measured at the same time points using the Hamilton Anxiety Rating Scale (HARS) [47], while the overall severity of psychiatric symptoms was investigated by the Clinical Global Impression-Severity Scale (CGI-S) [48] at baseline and endpoint.

Sleep disturbances were evaluated at all time points by the Pittsburgh Sleep Quality Index (PSQI) [49], using the cut-off total score of 5 to differentiate “good” from “bad” sleepers [50] and by the self-rated Visual Analogue Scale for sleep (VASs) [51].

Patients’ functioning and quality of life were assessed by clinicians at baseline and endpoint using the Functioning Assessment Short Test total scores (FAST) [52] and the Quality of Life Index (QL-I) [53].

Craving for alcohol was measured at baseline, 1 month, 3 months, and 6 months by the 14-item Obsessive Compulsive Drinking Scale total scores (OCDS) [54] and the self-rating instrument Visual Analogue Scale for craving (VASc) [51].

Abstinence from alcohol was documented by participants’ self-evaluation and a family member interview. It was further ascertained by blood alcohol concentration, hepatic indices such as aspartate transaminase (AST), alanine transaminase (ALT), gamma glutamyl-transferase (GGT), mean cellular volume (MCV), and blood carbohydrate-deficient transferrin (CDT) detected at each control.

The safety and tolerability of trazodone were confirmed by physical examination, repeated measurement of body weight, blood pressure, and pulse rate, routine laboratory, and instrumental clinical tests (hematology, chemistry, urinalysis, electrocardiographs with corrected QT interval calculation), and patients’ reports of any adverse events.

### 2.3 Statistical Analysis

Descriptive data were summarized as number and percentage (%) or mean ± standard deviation (M ± SD) for categorical and continuous variables, respectively. The outcome measures - the mean changes from baseline to 1, 3, and 6 months of each efficacy variable - were analyzed using a mixed model for repeated measurements (MMRM). Analyses were performed on all patients with at least one valid post-baseline assessment of the variables (full-analysis set, FAS). A significance level of 0.05 was used for each test. All analyses were conducted using IBM SPSS Statistics for Windows v. 25.0 (IBM Co., Armonk, New York, USA).

## 3 RESULTS

### 3.1 Demographic and Clinical Data

One hundred Caucasian subjects with MDD + AUD were included. About half of the sample (48%) reported lifetime substance abuse other than alcohol (cannabis: 18%, benzodiazepines: 15%, cocaine: 12%, opioids: 1%). The following additional pharmacotherapies were assumed throughout the treatment: mood stabilizers/anticonvulsants (valproate, gabapentin, topiramate), atypical antipsychotics (aripiprazole, olanzapine, quetiapine), benzodiazepines (diazepam, lorazepam), specific AUD medications, including acamprosate, nalmefene, and naltrexone.

Demographic, clinical, and baseline psychometric characteristics are summarized in Table **1**.

At the endpoint, data were available for 77 subjects. The drop-out rate was 23% (N = 23), of which 9 for relapse to heavy drinking; the total alcohol relapse rate (including minor relapses) was 31.1% (N = 24) of the final sample.

### 3.2 Primary Outcome Measures

Depressive symptoms, expressed as mean MADRS scores, improved during treatment with trazodone (F = 600, *p* < 0.001). Significant changes were already detected after one month of treatment and continued toward endpoint (Table **2**).

According to MADRS scores at endpoint, 42 patients (54.5%) were classified as “remitters”, 19 (24.7%) as “responders”, 7 (9.1%) as “non-responders”, and 9 (11.7%) as “relapsers”.

### 3.3 Secondary Outcome Measures

Improvements from baseline to endpoint were observed in the HARS (F = 952, *p <* 0.001), CGI-S (F = 222, *p <* 0.001), PSQI (F = 747, *p <* 0.001), VASs (F = 522, *p <* 0.001), FAST (F = 337, *p <* 0.001), and QL-I (F = 134, *p <* 0.001) scores (Table **2**).

At baseline, all patients reached the PSQI cut-off score for sleep disturbances and could be classified as “bad sleepers” (baseline score range: 8-17); at the endpoint, only 7.8% (n = 6) still reported bad quality of sleep (endpoint PSQI score range: 2-7).

All patients could be classified as at least “moderately ill” according to baseline CGI-S scores, and 42% of the initial sample was “markedly” or “severely ill” (CGI-S scores ≥ 5). At the endpoint, 58% of patients were classified as “Very Much Improved” (CGI-I score = 1).

Patients also displayed a reduction of craving symptoms, as measured by both OCDS (F = 118, *p <* 0.001) and VASc (F = 173, *p <* 0.001) scores (Table **2**).

### 3.4 Safety/Tolerability Profile

Emergent adverse events were detected within the first month of treatment in 25.8% of patients (*i.e*., dizziness/ drowsiness: 19.7%, dry mouth: 6.1%). At 3 months, dizziness/drowsiness was present in only 6.4% of patients and no side effect was reported at 6 months. No patient needed to discontinue treatment because of tolerability concerns.

Patients displayed significant reductions of MCV (95.1 ± 6.78 fl *vs*. 92.2 ± 5.71 fl, Z = 3.18, p = 0.001) and liver function tests at 6 months (AST: 26.2 ± 6.07 UI/L *vs.* 20.7 ± 4.48 UI/L, Z = 3.19, *p* = 0.001; ALT: 27.0 ± 9.35 UI/L *vs.* 21.5 ± 6.35 UI/L, Z = 3.2, p = 0.001; GGT: 42.5 ± 14.65 UI/L *vs.* 27.6 ± 7.65 UI/L, Z = 6.18, *p <* 0.001). No changes in QTc interval occurred from baseline to endpoint (405.4 ± 26.2 msec *vs.* 407.2 ± 26.26 msec, Z = -0.34, *p* = 0.73). A small but significant increase in weight was observed (66.4 ± 11.19 kg *vs.* 67.2 ± 10.97 kg, Z = -3.2, *p* = 0.002).

## 4 DISCUSSION

In this naturalistic, retrospective study, we observed that comorbid MDD + AUD patients displayed significant improvement in depressive symptoms following a six-month treatment with extended-release trazodone, with more than half of the sample showing clinical remission. A similar trend was observed in anxiety. Further, treatment with trazodone was associated with a marked improvement in sleep quality. Finally, we observed a reduction of craving symptoms over time as well as an improvement in global functioning and overall quality of life.

Management and treatment of the comorbidity between MDD and AUD are a concerning public health problem since both disorders reciprocally worsen clinical presentation and outcome. Previous studies on comorbid MDD + AUD provide empirical support for combined pharmacological approaches, where patients are prescribed both antidepressants and medications primarily used in the management of AUD, such as disulfiram and naltrexone [55]. Subsequent systematic analyses confirmed these findings and supported the use of antidepressants, although pointing out that the clinical relevance of treatment might be modest [9, 14]. This clinical population is often difficult to manage in real-world practice and, given the lack of comparative trials, the most effective approach is still uncertain [6].

Trazodone is a SARI antidepressant and is considered a “multifunctional” drug because of its multiple mechanisms of action [32]. Data from clinical trials suggest that the efficacy of trazodone is comparable to other classes of antidepressants, like SSRIs, SNRIs (serotonin-norepinephrine receptor inhibitors), and TCAs [56], and recent evidence supports a rapid-onset action, with significant improvements in depressive symptoms within the first weeks of treatment [57, 58]. Similarly, in our sample of patients with comorbid MDD + AUD, mood improvements were statistically significant after 1 month of treatment and became relevant at the endpoint [58].

Trazodone also shows anxiolytic and hypnotic properties, low cardiotoxicity, relatively mild side effects (the most frequent being headaches, dry mouth, fatigue, dizziness, and drowsiness/somnolence), and low potential for abuse. Specifically, extended-release trazodone ensures a gradual release of the drug all over the day, reducing both the plasma peak concentration and dosing frequency. Consequently, blood levels remain constantly above the minimum efficacious antidepressant concentration, and the absence of spikes is associated with lower peak dose adverse effects [34]. Administration of different formulations could help deliver more targeted interventions and, in turn, improve treatment tolerability and patient adherence [57]. Indeed, the characteristic pharmacokinetic profile of OAD trazodone makes it particularly useful in patients with moderate/severe depression, mild anxiety, and late insomnia [32]. Conversely, in light of the higher peak blood concentration, PR trazodone has been suggested in patients with mild/moderate depression, moderate-to-severe anxiety, and more marked difficulties in falling asleep [35].

Anxiety is a core symptom of MDD, associated with higher suicidal ideation, worse functioning, greater chronicity, and poor response to antidepressants [59]. It is also positively correlated with AUD. According to the self-medication theory of substance use, drinking to cope with negative affect (*e.g*., to reduce inner tension and negative feelings) is frequent in subjects with prominent anxiety features [60] and might transform into a persisting habit through negative reinforcement, *i.e*., reward generated by the reduction of a noxious stimulus [61, 62]. Higher amounts of alcohol and a fivefold risk of developing a persistent AUD in the following years have been reported in people who drink to cope with anxiety symptoms [62]. Further, in AUD patients, comorbid anxiety is associated with higher craving, which in turn increases the risk of drinking relapse and overall worse outcomes [10, 63]. Trazodone has proven beneficial effects on anxiety in MDD patients and has been widely used as an off-label treatment for generalized anxiety disorders [35, 36]. Also, a preliminary study has reported that treatment with trazodone has improved anxious symptoms in subjects with AUD [41].

Our study confirmed the effectiveness of extended-release trazodone in reducing anxiety in comorbid MDD + AUD patients, with significant improvements observed after the first month of treatment [58].

As for sleep disruption, extended-release trazodone has significantly improved sleep in this sample of MDD + AUD patients, further confirming its effectiveness in addressing insomnia in different clinical contexts.

Sleep disturbances are commonly described in up to 70% of depressive disorders [64]. Even when effective on mood symptoms, antidepressants do not necessarily improve sleep quality, and residual symptoms of insomnia have been reported after successful treatment of depression [65]. Further, MDD patients with comorbid insomnia are less likely to respond to antidepressant treatment and to achieve functional recovery [66].

Sleep disruptions are also common in AUD and insomnia is a significant predictor for psychopathology, including alcohol abuse [67]. Sleep disturbances have been described both during active drinking and withdrawal, are associated with clinical severity, depressive and anxiety symptoms, craving, and relapses, and have increasingly become a main target of treatment [40, 68]. Alcohol use might be a dysfunctional self-medicating strategy to address insomnia, leading to repeated drinking, tolerance, and alcohol abuse [69]. Indeed, the risk of developing AUD is higher in subjects with insomnia, especially when depressive symptoms are also present [9], and psychiatric burden mediates the relationship between the severity of alcohol use and sleep alterations [70]. Therefore, insomnia interventions could increase sleep quality and reduce symptoms of depression and anxiety in AUD patients [12, 71].

The use of trazodone to treat sleep alterations, especially in patients with psychiatric comorbidities, is a widespread strategy [72, 73] that yielded beneficial effects in MDD patients [35, 74] as well as on certain sleep measures and post-withdrawal insomnia in subjects with AUD [12, 75, 76]. Given that adding trazodone to first-line treatments for insomnia increased rates of remission in non-responding subjects [77] and the detrimental impact of sleep disruptions on the course of both MDD and AUD, the compound could represent a useful option to effectively address these features in comorbid patients.

In this study, we found a significant decrease in alcohol craving, both clinician- and self-rated, alongside the improvement of mood, anxiety, and sleep symptoms in comorbid MDD + AUD patients. Craving is a pivotal component of AUD, can be present during both active drinking and withdrawal, figures among the primary predictors of relapse [78], and should be specifically addressed by pharmacological and psychosocial interventions during AUD treatment [45, 79-81]. Further, craving may reciprocally interact with other symptoms, *i.e*., negative affect, anxiety, and insomnia, leading to increased clinical severity and detrimental outcomes [71, 82, 83]. Preliminary observations reported some effect of trazodone in reducing craving in AUD patients [41, 84]. Although more studies are needed to confirm these findings, our results further support the usefulness of extended-release trazodone in this clinical population.

Here, we also observed that extended-release trazodone has improved measures of functioning and quality of life. Both depression and alcohol abuse are associated with poor psychosocial and occupational functioning, with a negative impact on quality of life, requiring increasing attention on functional recovery and quality of life as a main target of therapy.

Finally, extended-release trazodone showed a favorable safety and tolerability profile in our sample. About ¼ of patients reported mild side effects after 1 month of treatment, which progressively disappeared at the endpoint. Significant improvements in MCV and liver function tests were measured during treatment, possibly reflecting the reduction of alcohol intake. An increase in body weight was observed at the endpoint, although of a modest entity and without medical implications.

Some limitations must be acknowledged for this study: the retrospective nature of our observations, the relatively small sample size, the lack of a control group, the reliability of the self-administered questionnaires, and the concomitant additional treatments that might have contributed to our findings and limit their generalizability. Further studies involving larger samples and control groups are certainly needed to replicate these results.

## CONCLUSION

The comorbidity of MDD and AUD is complex in terms of both assessment and treatment and yields a great burden on the healthcare system because of increased severity, chronicity, reduced functioning, and generally unfavorable outcomes. Despite the increasing prevalence, patients with alcohol/substance use comorbidity are usually excluded from registration trials; therefore, a study including MDD patients with co-occurring AUD can more closely relate the results to real-world practice. Although several studies tried so far to summarize available findings regarding evidence-based medications and psychosocial interventions to better inform clinical approaches, improvements in treatment for this population are still needed. Patients with comorbid MDD and AUD are usually burdened by several diverse and overlapping features, including higher rates of anxiety, psychomotor agitation, and insomnia, which might be particularly intense in certain phases, *i.e*., withdrawal. These symptoms interact reciprocally and with additional features like craving, increasing the risk for drinking relapses, worsening overall severity, and often requiring combined psychopharmacological approaches to be properly managed. The heterogeneity of comorbid MDD + AUD and key differences within subpopulations could inform personalized, more targeted interventions to improve outcomes. Trazodone has proven to be a valid antidepressant, particularly useful in patients with significant anxiety and insomnia. Also, it appears to exert a beneficial effect on craving. Taken together, these findings suggest that extended-release trazodone might represent a useful pharmacological option in addressing MDD + AUD comorbidity.

## Figures and Tables

**Table 1:** Sociodemographic and clinical characteristics at baseline.

	**N %; M ± SD**
**Overall**	100
**Sociodemographic Features**	
Age (years)	47.4 ± 12.5
**Gender**	
Male Female	57 (57) 43 (43)
Education level (years)	13.9 ± 3.7
Occupation (employed)	65 (65)
Marital status (married)	22 (22)
**Clinical Data**	
Age of onset (years)	34.6 ± 13
Duration of illness (years)	12.7 ± 8.7
Number of lifetime episodes	3.1 ± 1.8
Duration of the current episode (months)	3.7 ± 0.6
Suicide attempts	10 (10)
Trazodone dose (mg/day)	192 ± 67.8
**Other Psychopharmacotherapy** Mood stabilizers/anticonvulsants Atypical antipsychotics Sedative-hypnotics/anxiolytics Anti-craving drugs	66 (66) 51 (51) 12 (12) 17 (17) 41 (41)
Family history of psychiatric disorders	61 (61)
Medical comorbidities	28 (28)
Smoking habits	70 (70)
BMI	23.6 ± 3.6
**Psychometric Assessment**	
MADRS	27.4 ± 2.02
HARS	23.8 ± 3.9
CGI-S	4.4 ± 0.5
PSQI	12.8 ± 2.1
VASs	6.7 ± 0.6
FAST	46.4 ± 6.8
QL-I	5.3 ± 0.6
OCDS	21.9 ± 8.9
VASc	6.9 ± 1.6

**Abbreviations:** BMI: Body Mass Index, CGI: Clinical Global Impression, FAST: Functioning Assessment Short Test, HARS: Hamilton Anxiety Rating Scale, M: Mean, MADRS: Montgomery-Åsberg Depression Rating Scale, OCDS: Obsessive Compulsive Drinking Scale, PSQI: Pittsburgh Sleep Quality Index, QL-I: Quality of Life Index, SD: Standard Deviation, VASc: Visual Analogue Scale for craving, VASs: Visual Analogue Scale for sleep.

**Table 2:** Psychometric evaluation at selected time-points (MMRM, FAS).

		**Change from Baseline**				
	**Mean ± SD**	**Mean ± SE**	**95% Confidence Interval**		***t***	***p***
			**Lower**	**Upper**		
**Psychiatric Symptoms**						
MADRS 1 m 3 m 6 m	24.2 ± 1.98 14.4 ± 2.75 8.68 ± 4.95	-3.41 ± 0.48 -12.9 ± 0.49 -18.8 ± 0.49	-4.37 -13.91 -19.7	-2.47 -11.97 -17.77	-7.04 -26.15 -38.09	< 0.001 < 0.001 < 0.001
HARS 1 m 3 m 6 m	20.05 ± 3.1 14.09 ± 2.84 9.04 ± 2.18	-3.57 ± 0.29 -9.36 ± 0.30 -15.19 ± 0.31	-4.15 -9.95 -15.79	-2.98 -8.77 -14.6	-12 -31 -49.9	< 0.001 < 0.001 < 0.001
CGI-S 6 m	2.35 ± 0.53	-2.04 ± 0.04	-2.13	-1.96	-47.1	< 0.001
**Sleep Quality**						
PSQI 1 m 3 m 6 m	10.4 ± 1.86 7.08 ± 1.43 4.12 ± 1.41	-2.4 ± 0.33 -5.7 ± 0.34 -8.89 ± 0.35	-2.9 -6.29 -9.48	-2.02 -5.4 -8.38	-12.6 -29.1 -44.4	< 0.001 < 0.001 < 0.001
VASs 1 m 3 m 6 m	5.74 ± 0.74 4.14 ± 0.95 2.15 ± 1.17	-1.02 ± 0.24 -2.59 ± 0.25 -4.57 ± 0.26	-1.21 -2.83 -4.81	-0.79 -2.41 -4.39	-9.4 -24.6 -42.69	< 0.001 < 0.001 < 0.001
**Measures of Craving**						
OCDS 1 m 3 m 6 m	18.4 ± 6.01 12 ± 4.18 6.29 ± 3.26	-3.99 ± 1.95 -10.4 ± 1.85 -16.11 ± 1.99	-5.72 -12.2 -17.91	-2.19 -8.6 -12.3	-4.33 -11.28 -17.47	< 0.001 < 0.001 < 0.001
VASc 1 m 3 m 6 m	6.22 ± 1.18 4.56 ± 1.15 2.5 ± 1.43	-0.77 ± 0.20 -2.43 ± 0.21 -4.48 ± 0.22	-1.18 -2.84 -4.89	-0.37 -2.02 -4.06	-3.72 -11.69 -21.07	0.03 < 0.001 < 0.001
**Clinician-rated Functioning**						
FAST 6 m	27.5 ± 5.32	-19 ± 1.08	-20.9	-17	-19.1	< 0.001
**Quality of Life**						
QL-I 6 m	6.79 ± 0.51	1.56 ± 0.1	1.3	1.83	11.6	< 0.001

**Abbreviations:** CGI-S: Clinical Global Impression - Severity Scale, FAST: Functional Assessment Short Test, HARS: Hamilton Anxiety Rating Scale, MADRS: Montgomery-Åsberg Depression Rating Scale, OCDS: Obsessive Compulsive Drinking Scale, p: p-value, PSQI: Pittsburgh Sleep Quality Index, QL-I: Quality of Life Index, SD: standard deviation, SE: standard error, VASc: Visual Analogue Scale for craving, VASs: Visual Analogue scale for sleep.

## Data Availability

Not applicable.

## LIST OF ABBREVIATIONS

ALT: Alanine Transaminase
AST: Aspartate Transaminase
AUD: Alcohol Use Disorder
CDT: Carbohydrate-deficient Transferrin
GGT: Gamma Glutamyl-transferase
MDD: Major Depressive Disorder
PTSD: Post-traumatic Stress Disorder
RCTs: Randomized Clinical Trials
rTMS: Transcranial Magnetic Stimulation
SARI: Serotonin Antagonist and Reuptake Inhibitor
SSRIs: Selective Serotonin Reuptake Inhibitors
SUD: Substance Use Disorder
TCAs: Tricyclic Antidepressants
tDCS: Transcranial Direct Current Stimulation
